# Supplementary material for: Tuck-KGC: based on tensor decomposition for diabetes knowledge graph completion model integrating Chinese and Western medicine
Source: PeerJ Comput Sci. 2025 Feb 19;11:e2522. doi: 10.7717/peerj-cs.2522 (PMC11888843; doi:10.7717/peerj-cs.2522)
Supplement: Appendix S1 [file peerj-cs-11-2522-s001.pdf]

Appendix A

Referring to the characteristics of traditional Chinese and Western medicine diagnosis and treatment, TCM syndrome differentiation is combined with Western medicine disease differentiation. Syndrome differentiation refers to summarizing symptoms and then determining treatment methods and specific prescriptions based on symptoms; disease differentiation refers to deducing the disease from symptoms, and then the relevant syndromes are derived from the disease, and finally the treatment methods and specific prescriptions are determined by the syndromes. According to the characteristics of diabetes, combined with the relationship between the biochemical test indicators of Western medicine and the disease, as well as the three classifications of diabetes in Traditional Chinese Medicine, at the entity concept level, it is divided according to the "Ten High and One Low" of Traditional Chinese Medicine and the classification definition of Western Medicine. There are twelve specific categories and their relationships. See Table A1, Table A2, Table A3.

Table A1：Common entity categories of Chinese and Western medicine and their explanations

Entity type	Type explanation
Cause	Causes and risk factors of disease
Clinical manifestations	Symptoms and signs
Drug	Routine medication
Medication frequency	Medication frequency
Duration	Duration of symptoms and duration of medication
Storage	storage method
Medication method	time, oral, injection, etc.
non-drug treatment	radiotherapy, traditional Chinese medicine treatment
Degree	severity of illness
Adverse reactions	Adverse reactions after treatment
Part	body part, biological tissue
Dosage	usage amount

Table A2： Western medicine entity categories and their explanations

Entity type	Type explanation
Medicine property	Traditional Chinese Medicine’s Understanding of Drugs
Source	Source
Meridian tropism	Part of the body where the drug acts
Chinese prescription	Traditional Chinese Medicine Treatment Prescriptions
Chinese treatment	Traditional Chinese Medicine Treatment
Addition and subtraction	Medication adjustment

Table A3：Traditional Chinese Medicine entity categories and their explanations

Entity type	Type explanation
Inspection Method	laboratory tests, Film degree exam
Biochemical test indicators	Indicator value
OCT type	Prescription, Non-prescription drugs
Drug regulatory classification	Level 1, 2, and 3
